# Limonene and carvacrol combined with drugs that inhibit ergosterol synthesis are potent against *Leishmania major*

**DOI:** 10.1007/s00210-026-05160-9

**Published:** 2026-03-16

**Authors:** Rita de Cássia Viana de Carvalho, Karla Germana dos Reis Bacelar, Eduardo Lima Pereira, Bianca Soriano dos Anjos, Evellyn Caroline Silva Melo, Francisco das Chagas de Souza Cunha, Paulo Sérgio de Araujo Sousa, Jefferson Almeida Rocha, Leiz Maria Costa Véras, Klinger Antônio da Franca Rodrigues, Maria das Graças de Freire Medeiros, Daniel Dias Rufino Arcanjo, Michel Mualem de Moraes Alves, Fernando Aécio de Amorim Carvalho

**Affiliations:** 1 BioLeish (Advanced Research in Leishmania and Alternative Models), Medicinal Plants Research Center, Campus Ministro Petrônio Portella, Federal University of Piauí, TeresinaPiauí, 64049-550 Brazil; 2 Medicinal Chemistry and Biotechnology Research Group, QUIMEBIO, Federal University of Maranhão, UFMA, São Bernardo, Maranhão, MA Brazil; 3 CCB (Bacabal Science Center), Federal University of Maranhão (UFMA), Bacabal Campus, BacabalMaranhão, 65700-000 Brazil; 4 CEBSI (Special Coordination of Biological and Health Sciences), Federal University of Maranhão, São LuísMaranhão, 65080805 Brazil; 5 Department of Pharmacy, Federal University of Piaui, TeresinaPiauí, 64049-550 Brazil; 6 Department of Biophysics and Physiology, Federal University of Piaui, TeresinaPiauí, 64049-550 Brazil; 7 Graduate Program in Technologies Applied to Animals of Regional Interest, Federal University of Piauí, TeresinaPiauí, 64049-550 Brazil; 8 Department of Biochemistry and Pharmacology, Federal University of Piauí, TeresinaPiauí, 64049-550 Brazil; 9 Department of Veterinary Morphophysiology, Federal University of Piauí, Campus Ministro Petrônio Portella, Teresina, PI 64049-550 Brazil

**Keywords:** Monoterpenes, Nystatin, Leishmaniasis, Polyene antifungals, Drug synergy

## Abstract

Leishmaniases are neglected diseases widely distributed in tropical and subtropical countries, caused by parasites of the *Leishmania* genus. Current therapy includes pentavalent antimonial and amphotericin B, but adverse effects persist. The development of a new, more selective, and less toxic combination therapy is therefore a rational and promising approach. In this study, we report the effects of combinations of the monoterpenes limonene (Lim) and carvacrol (Car) with drugs targeting ergosterol biosynthesis, namely nystatin (Nys), tioconazole (Tio), and rosuvastatin (Ros), on *Leishmania major*, their cytotoxicity on macrophages, and we evaluate their synergism and mechanisms of action. The combinations inhibited the growth of *L. major* promastigotes, with Lim-Car combined with nystatin (3:2) exhibiting the highest activity, showing an IC_50_ of 2.02 µg/mL. Furthermore, this combination demonstrated greater action against amastigotes (IC_50_ of 0.53 µg/mL) and a high selectivity index (33.60). Low cytotoxicity was observed in murine macrophages (CC_50_ 17.81 µg/mL), as well as a low hemolytic potential (CH_50_ of > 100 µg/mL). The Lim-Car and nystatin (3:2) combination presented a synergistic effect, with a fractional inhibitory concentration index of 0.4. Changes in parasite membrane integrity were also observed, which may be linked to the interaction of nystatin with the enzyme 14-alpha demethylase, along with an increase in TNF-α expression and a reduction in IL-10 and IL-6 levels. These results suggest that the combination of Lim-Car with Nys (3:2) may exert a summative effect through multiple biochemical pathways and warrants further investigation as a potential combined therapy with antileishmanial activity.

## Introduction

Leishmaniases are neglected parasitic diseases that represent a serious public health problem and are caused by parasites of the genus *Leishmania* that infect different vertebrates and are predominant in intertropical regions (Akbari et al. 2021). Brazil stands out for having a high incidence of different forms of the disease, with the most prevalent being the cutaneous form (CL) with clinical manifestations that vary from a simple lesion that can heal spontaneously or multiple nodules spread throughout the body and even the development of the mucocutaneous form, reaching the palate and nose (Brannigan and Wilkinson 2021).

According to the WHO, CL is classified as one of the six infectious diseases with the greatest impact due to its high detection coefficient and the ability to produce deformities (World Health Organization 2010). Therefore, it is necessary to increase the therapeutic arsenal related to leishmaniasis since the most used drugs are still pentavalent antimonial and amphotericin B, which cause a series of adverse effects, with a long treatment period, which makes it expensive and, in addition, is associated with parasite resistance (Gazanion et al. 2016).

Therefore, over the years, the need for the development of new drugs has been observed, while several resistance mechanisms of the parasite have been reported, which could prevent the emergence of one of the main obstacles to leishmania infectious therapy (Armijos et al. 2004). Considering all the problems surrounding antileishmanial drugs, even though they lead to a therapy with greater effectiveness and low adverse effects, there are still unknown limitations, including the immunological characteristics of each individual, which can become a limiting factor in treatment (Hendrickx et al. 2019).

With this in mind, new strategies in the search for more selective drugs for the parasite and with less toxicity have been established, such as natural products associated with synthetic drugs, which present many opportunities. Limonene and carvacrol are monoterpenes found in several plants and demonstrate a variety of biological properties, especially proven leishmanicidal action. In addition to rosuvastatin, used in hypercholesterolemia in humans, and the antifungals tioconazole and nystatin, which act on the biochemical pathway of ergosterol, the main component of the leishmania membrane, an interesting fact is that combined with monoterpenes can present optimized biological effects (Carvalho et al. 2021).

Therefore, considering the importance of obtaining new drugs that may be an alternative to the treatment of leishmaniasis, the objective of this study was to evaluate the biological activity of the combination of limonene and carvacrol associated with the drugs rosuvastatin, nystatin, and tioconazole, against promastigote and amastigote forms of *Leishmania major*, in addition to the cytotoxic and immunomodulatory activity in murine macrophages, as well as evaluation of membrane integrity, evaluation of cytokine production, and molecular docking.

## Material and methods

### Substances used

Dimethyl sulfoxide (DMSO: 99%) was purchased from Merck Chemical Company (Germany). Schneider culture medium, RPMI medium, fetal bovine serum (FBS), MTT (3-(4,5-dimethylthiazol-2-yl)−2,5-diphenyltetrazolium bromide), Alamar Blue (Resazurin®), and the antibiotics penicillin and streptomycin was purchased from Sigma Chemical (Sigma-Aldrich Brazil). The antibiotic amphotericin B (Amp B 90%) and rapid panoptic® were purchased from Cristália (São Paulo, SP). The substances limonene and carvacrol (Sigma-Aldrich, Vienna, Austria) had a purity of 97% and 98%, respectively. Nystatin (Nys), rosuvastatin (Ros), and tioconazole (Tio) (Sigma Chemical Co, St Louis, USA) originated the mixture of monoterpenes [Lim-Car (4:1)] with the aforementioned drugs; the combinations were carried out in the proportions for in vitro (1:4, 2:3, 3:2, 4:1, and 1:1). Our experiments were based on the work developed by Pastor et al. (2015). Combinations were diluted in DMSO at a concentration of 10 mg/mL for the experiments.

### Parasites and cells

*Leishmania major* (MHOM/IL/80/Friendlin) was used to evaluate the antileishmanial activity. Promastigotes were cultivated in Schneider’s medium supplemented with 10% FBS, 100 U/mL penicillin, and 100 µg/mL streptomycin, incubated in a B.O.D. incubator at 26 °C. Murine macrophages were collected from the peritoneal cavities of BALB/c mice after previous elicitation (72 h) by intraperitoneal application of 2 mL of 3% thioglycolate; sheep blood (9 months old) was collected from the Agricultural Sciences Center (CCA/UFPI) located in Teresina, PI, Brazil. All protocols were approved by the Animal Research Ethics Committee (CEEUA nº 707/21).

### Antileishmanial activity of the Lim-car (4:1) combination with drugs Nys, Ros, and Tio on promastigote forms of *L. major*

The test was performed in 96-well cell culture plates with 1 × 10^6^ promastigotes of *L. major* in logarithmic growth phase/100 µL of Schneider’s medium. Subsequently, the different combinations of Lim-Car (4:1) with Nys, Ros, and Tio were added to the wells in triplicate and serial dilutions were performed, reaching eight ranges of desired final concentrations (0.78–100.00 µg/mL). Then, the plate was incubated at 26 °C in a B.O.D. incubator for 48 h, and 6 h before the end of this period, 20 µL of resazurin 1 × 10^−3^ mol/L was added, and then incubated again. At the end, the absorbance plates were read at 550 nm. For the negative control, Schneider’s medium was added, and for the positive control, amphotericin B (2 µg/mL) (Carneiro et al. 2012).

### Assessment of cytotoxicity

#### Cytotoxicity in macrophages and selectivity index (SI)

Lim-Car (4:1) combinations with Nys, Ros, and Tio were evaluated using the MTT assay. In a 96-well plate, 2 × 10^5^ macrophages per well were incubated in 100 µL of RPMI medium (supplemented with 10% FBS, 10,000 IU penicillin and 1000 IU streptomycin) in an incubator at 37 °C and 5% CO_2_ for 4 h. After that, the combinations were added in serial concentrations and eight final concentration ranges were attained (0.78–100.00 µg/mL), and incubated at 37 °C and 5% CO_2_ for 48 h. After this period, cytotoxicity was evaluated by adding 10% MTT [5 mg/mL]. Later, the supernatant was discarded and 100 µL of DMSO was added. Finally, the absorbance (550 nm) was measured. The selectivity index of each treatment was calculated by dividing the mean cytotoxic concentration (CC_50_) observed by the mean inhibitory concentration (IC_50_) (Alves et al. 2017).

#### Cytotoxicity in red blood cells

In the hemolytic evaluation tests, 5 mL of sheep’s blood was diluted in 80 µL of PBS (hematocrit concentration to 5% of red blood cells). Compounds of Lim-Car (4:1) combination with Nys, Ros, and Tio were added to the blood compound, and eight final concentration ranges were attained (0.78–100.00 µg/mL), diluted in 20 µL of PBS. Then, the mixture was incubated at 37 °C for 1 h, and later, 200 µL of PBS was added to it, and later, the mixture was read at 550 nm. The controls were defined as a negative control for non-occurrence of hemolysis (PBS) and a positive control for 100% hemolysis (Milli-Q water). The results of the evaluation were defined as mean hemolytic concentration (CH_50_) and 100% hemolysis for the positive control; the values are given in % (Dias et al. 2013).

#### Activity of the Lim-car 4:1 combination with nystatin, rosuvastatin, and tioconazole in macrophages infected with m-car 4:1 combination with nystatin, rosuvastatin, and tioconazole in macrophages infected with m-car 4:1 combination with nystatin, rosuvastatin, and tioconazole in macrophages infected with *L. major*

From the step described above, the following protocols were carried out with compounds for each of the combinations that exhibited greater antileishmanial activity and lower cytotoxicity in macrophages.

In 24-well culture plates, macrophages were added at a concentration of 2 × 10^5^ cells per well in 500 µL of supplemented RPMI, along with sterile coverslips of 13 mm in diameter. These plates were incubated at 37 °C in 5% CO_2_ for 4 h for macrophage adherence. The attached macrophages were then incubated with *L. major* promastigotes (in stationary phase) containing a ratio of 10 promastigotes for each macrophage in 5% CO_2_ at 37 °C for 4 h. After that, the infected culture was incubated with values corresponding to ½ × IC_50_, 1 × IC_50_, and 2 × IC_50_ of each compound on the promastigote forms and amphotericin at a concentration of 0.62 µg/mL. After this period, the coverslips were removed from the wells and staining was performed using rapid panoptic®. For each treatment, the number of infected macrophages and the parasite load (survival index of amastigotes) were counted by going through the compound fields until reaching a count of 100 macrophages, using optical microscopy (Carneiro et al. 2012).

### Investigation of synergistic combinations

The fractional inhibitory concentration (FIC) was calculated according to Johnson et al. (2004) using the following formula: FIC index = [A]/IC_50_A + [B]/IC_50_B, where IC_50_A and IC_50_B are the IC_50_ values on the amastigotes of each monoterpene tested alone and [A] and [B] are the IC_50_ values on the amastigotes of monoterpenes A or B when the treatment was performed in combination. An index less than or equal to 0.5 indicates synergism, while an index greater than 4 indicates antagonism. A FIC index between 0.5 and 4 indicates indifference. An isobologram was constructed for antileishmanial activity, representing the standard error of the mean for each component of the Lim-Car combination.

### Evaluation of macrophage activation parameters

#### Evaluation of lysosomal activity

In 96-well plates, 2 × 10^5^ of peritoneal macrophages was plated per well and incubated at 37 °C and 5% of CO_2_ with the compounds, through serial dilutions attaining five ranges of final concentrations (6.25–100.00 µg/mL). After 48 h, 10 µL of neutral red solution in 2% DMSO® was added and incubated for 30 min. The wells were washed with 0.9% saline at 37 °C, which led to the process of solubilization of the neutral red (Sigma Chemical Co, St Louis, USA) of the lysosomal vesicles; 100 µL of the extraction solution was added. And after 30 min, the absorbance was read at 550 nm (Alves et al. 2017).

### Determination of phagocytic capacity

Macrophages were grown as described in the previous item. After 48 h of incubation, 10 µL of stained zymosan (Sigma Chemical Co, St Louis, USA) solution was added to the compounds and again incubated at 37 °C for 30 min; later, 100 µL of Baker fixative was added to block phagocytosis. A 0.9% saline solution was used at this time to remove the zymosan and neutral red not phagocytized. Then, the supernatant was removed, and 100 µL of extraction solution was added, and the absorbance reading was performed at 550 nm (Alves et al. 2017).

### Evaluation of induction nitric oxide synthesis

Macrophages were grown as described in the previous item in the presence or absence of promastigote of *L. major*. After 24 h of incubation, the supernatants from the cell culture were transferred to another 96-well plate for the dosage of nitrite. The standard curve was prepared with sodium nitrite in RPMI medium at varying concentrations of 1, 5, 10, 25, and 50 µM. At the time of dosage, equal parts of the compounds (supernatants) or the solutions prepared to obtain the standard curve were mixed with the same volume of Griess® reagent (Sigma Chemical Co, St Louis, USA) (1% Sulfanilamide in H_3_PO_4_ 10% (v:v) in ultrapure water, added in parts equal to 0.1% naphthylenediamine in ultrapure water) and the absorbances were read at 550 nm; the result was plotted as a percentage of nitrite production (Carneiro et al. 2012).

### Membrane integrity assay

Plasma membrane integrity in promastigote forms of *L. major* treated with nystatin and Lim-Car 4:1 combination with Nystatin was evaluated using the SYTOX green dye method (Thermo Fisher Scientific, Eugene, OR, USA). Promastigote forms in logarithmic growth were seeded in Schneider medium and exposed to the nystatin and Lim-Car 4:1 combination with nystatin at concentrations corresponding to IC_50_, 2 × IC_50_, and 4 × IC_50_ in the presence of SYTOX green dye (5 µM) for 7 h at 26 °C in a BOD incubator. The plates were then read in a fluorescence reader with an emission filter of 523 nm and excitation at 488 nm (Sousa et al. 2023).

### Assessment of cytokine production

According to Rodrigues et al. (2021), to carry out cytokine evaluation, the supernatant saved after the murine macrophage infection assay was used to evaluate cytokine production. Analysis kits (optEIATM ELISA, Pharmingen, San Diego, CA, USA) were used following the manufacturer’s protocol to examine the release of cytokines (TNF-α, IL-12, IL-10, IL-6), and standard recombinant cytokines were used to produce a curve. The reading was performed in a spectrophotometer at 450 nm.

### Molecular docking

The protein trypanothione reductase and 14-alpha demethylase (CYP51) were prepared using CHIMERA software version 13.1, where all water molecules, ions, and residues that do not form part of the macromolecule structure were removed from the structure. The three-dimensional (3D) structure of nystatin was designed and optimized using GaussView 5 and Gaussian 09w software, respectively. The optimization was carried out employing the density functional theory (DFT) method with the hybrid B3LYP functional and the STO-3G basis set (Sousa et al. 2024).

The 3D structure of protein trypanothione reductase and 14-alpha demethylase (CYP51) was obtained from the Protein Data Bank (PDB) with the code 2JK6 and 3L4D, respectively. All molecular docking procedures were conducted using the AutoDock 4.2 package. Proteins and ligands were prepared for molecular interaction simulations with AutoDockTools. Gasteiger partial charges were calculated after the addition of polar hydrogens, and non-polar hydrogen atoms from the protein and ligands were subsequently added. A cubic box of 60 × 60 × 60 Å was generated at the protein active site to delineate the interaction site of the ligand with the protein. Other docking parameters were set as default values, and the molecular dockings were performed using AutoDock Vina. For a more detailed analysis, the complex with the best obtained binding energy was analyzed using the Chimera and BIOVIA Discovery Studio Visualizer software (Oliveira et al. 2022; Sousa et al. 2023, 2024; Nogueira et al. 2024; Araújo et al. 2024; Nascimento et al. 2024).

### Statistics

All trials were performed in triplicate in three independent experiments. The mean inhibitory concentration (IC_50_), mean cytotoxic concentration (CC_50_), and mean hemolytic concentration (CH_50_) with 95% confidence limit were calculated using probit regression. The selectivity index was calculated by dividing CC_50_ by IC_50_. Analysis of variance (ANOVA) followed by Bonferroni’s test was performed, taking the value of *p* < 0.05 as the maximum level of statistical significance.

## Results and discussion

In this study, the effects of associations of the monoterpenes limonene (Lim) and carvacrol (Car) in a ratio of 4:1, respectively [Lim-Car (4:1)], combined with the drugs nystatin (Nys), tioconazole (Tio), and rosuvastatin (Ros), were characterized in different proportions on promastigotes and intracellular amastigotes and in cytotoxicity assays, as well as macrophage activation studies, membrane integrity evaluation, and molecular docking. It has already been demonstrated that the Lim-Car association (4:1) has leishmanicidal action (Carvalho et al. 2021), and when combined with drugs targeting ergosterol biosynthesis, it showed potent activity against *L. major.* All tested combinations reduced the IC_50_ value compared to the isolated substances. In particular, the combination of Lim-Car (4:1) with nystatin at a 3:2 ratio exhibited the lowest IC_50_ value (2.02 ± 0.8 µg/mL), being approximately twice as effective as most other combinations tested (Table 1).

**Table 1:** Antileishmanial activity against promastigotes of *L. major* of limcar, rosuvastatin, nystatin, tioconazole, and the results of combinations between the substances studied

Substances	4:1 limcar	Rosuvastatin	Nystatin	Tioconazole	Amp B
Promastigote *L. major* IC_50_ µg/mL^a^	15.40 ± 0.23	5.11 ± 0.01	5.16 ± 0.09	5.91 ± 0.07	0.62 ± 0.14
Substances	**Limcar + Ros 1:4**	**Limcar + Ros 2:3**	**Limcar + Ros 3:2**	**Limcar + Ros 4:1**	**Limcar + Ros 1:1**
Promastigote *L. major* IC_50_ µg/mL^a^	4.16 ± 0.12	5.45 ± 013	4.62 ± 0.13	2.96 ± 0.10	4.52 ± 0.11
Substances	Limcar + Nys 1:4	Limcar + Nys 2:3	Limcar + Nys 3:2	Limcar + Nys 4:1	Limcar + Nys 1:1
Promastigote *L. major* IC_50_ µg/mL^a^	4.91 ± 0.11	2.83 ± 0.9	2.02 ± 0.8	4.39 ± 0.14	4.17 ± 0.12
Substances	Limcar + Tio 1:4	Limcar + Tio 2:3	Limcar + Tio 3:2	Limcar + Tio 4:1	Limcar + Tio 1:1
Promastigote *L. major* IC_50_ µg/mL^a^	2.21 ± 0.11	3.85 ± 0.12	4.91 ± 0.13	5.98 ± 0.14	3.31 ± 0.12

^a^Concentration of the drug that causes 50% mortality. Values represent the mean IC_50_ calculated in at least three independent experiments ± standard error of the mean. For this calculation, the probit regression model was used

In the search for new drugs for leishmaniasis therapy, biochemical pathways vital to the parasite are potential targets. In this context, drugs that act on the sterol biosynthetic pathway, specifically on the synthesis of ergosterol because this molecule is an important component of the leishmania membrane, become promising leishmanicidal agents. Furthermore, the combination of these drugs with bioactive molecules can be an advance in terms of treating the disease, as there are numerous advantages of combined therapy, from synergism to even reduced toxicity (Yamamoto et al. 2015).

It is noteworthy that drug combinations, as well as drug repositioning, hold significant importance due to their beneficial effects in therapy. The best result among the combinations evaluated in this study against *L. major* promastigotes was the ratio of 3 parts Lim-Car (4:1) to 2 parts nystatin [Lim-Car (4:1) + Nys] (3:2). The Lim-Car (4:1) combination alone had an IC_50_ of 15.40 µg/mL (Table 1), but when associated with nystatin at a 3:2 ratio, the IC_50_ value was reduced to 13.12%. These findings may contribute to toxicity reduction since decreasing the therapeutic dose of a specific drug likely translates to a reduction in its use-related toxicity (Seifert et al. 2007).

Given the promising results obtained for the antileishmanial activity of the Lim-Car (4:1) combination with the drugs, the compounds that demonstrated the highest antileishmanial activity were selected and evaluated for their cytotoxicity in murine macrophages, as well as their hemolytic activity in sheep erythrocytes. In the macrophage viability assay, Nys, Ros, and Tio exhibited average cytotoxic concentration (CC_50_) values of 55.04 µg/mL, 171.92 µg/mL, and 28.48 µg/mL, respectively, while all the combinations tested showed higher cytotoxicity, with CC_50_ values ranging from 14.25 to 17.81 µg/mL (Table 2). Regarding macrophage cytotoxicity, all compounds were less toxic than amphotericin B (Amp B) (CC_50_ = 6.15 µg/mL (Table 2).

**Table 2:** Antileishmanial activity, cytotoxic effect on mammalian cells, and selectivity index values calculated for limcar, rosuvastatin, nystatin, tioconazole, and combinations

Substances	Macrophages		Internalized amastigotes		Erythrocytes		Selectivity index
	CC_50_ µg/mL^a^		IC_50_ µg/mL^b^		CH_50_ µg/mL^c^		SI^e^
4:1 Lim-Car	176 ± 0.07	5.54 ± 1.64		> 100		31.7	
Rosuvastatin	171.92 ± 0.02	1.98 ± 1.05		> 100		16.98	
Nystatin	55.04 ± 0.01	1.80 ± 1.36		> 100		30.57	
Tioconazole	28.48 ± 0.13	2.0 ± 0.81		> 100		14.24	
Limcar^d^ + Ros 2:3	16.50 ± 0.14	1.30 ± 1.94		> 100		12.69	
Limcar + Nys 3:2	17.81 ± 0.15	0.53 ± 2.09		> 100		33.60	
Limcar + Tio 1:4	14.25 ± 0.15	0.76 ± 2.47		> 100		18.75	
Amp B	6.15 ± 0.24	0.39 ± 0.01		231.9 ± 16.39		15.76	

^a^Cytotoxic concentration of the drug that causes 50% of macrophage mortality

^b^Concentration of the drug that causes 50% mortality

^c^Hemolytic concentration 50%

^d^Limcar = Lim + car (4:1)

^e^SI = CC_50_/IC_50_ (against internalized amastigotes)

Although the Lim-Car (4:1) combinations with the tested drugs showed higher toxicity compared to the isolated substances, this effect was only observed at higher concentrations. This analysis highlights the importance of drugs targeting sterol synthesis, as they may exhibit high selectivity for parasites without causing cellular damage (Santiago-Silva et al. 2022).

The strategy of combination therapy has been investigated for drugs used in leishmaniasis infections, showing that lower drug doses can be employed without compromising efficacy while reducing toxicity. In general, combination studies propose reducing drug doses as well as treatment duration, which potentially improves therapy adherence due to lower toxicity, in addition to cost reduction and other benefits (Uliana et al. 2018).

Another alternative method for evaluating in vitro toxicity is through the use of erythrocytes. None of the evaluated compounds exhibited significant hemolytic effects, with CH_50_ values for all compounds > 100 µg/mL (Table 2).

The antileishmanial activity and cytotoxicity assays were essential to select the best compounds and evaluate the interaction of Lim-Car (4:1) with the drugs Ros, Nys, and Tio in intracellular amastigotes to assess the effect of combinations, mimicking the infection of a vertebrate host cell in vitro. The results obtained were superior compared to tests on promastigote forms, with a reduction in IC_50_ for all evaluated compounds, ranging from 0.53 to 1.98 µg/mL (Table 2), demonstrating greater efficacy against *L. major* amastigotes. The combination [Lim-Car (4:1) + Nys] (3:2) again showed the best results, with an IC_50_ = 0.53 µg/mL (Table 2). Thus, our results suggest a higher vulnerability of amastigotes to this combination, possibly due to improved synergistic properties for this evolutionary stage of *Leishmania* or a contribution of metabolic pathway activation in the adopted model through the action of the combination of natural products and drugs (Intakhan et al. 2022).

**Fig. 1:**
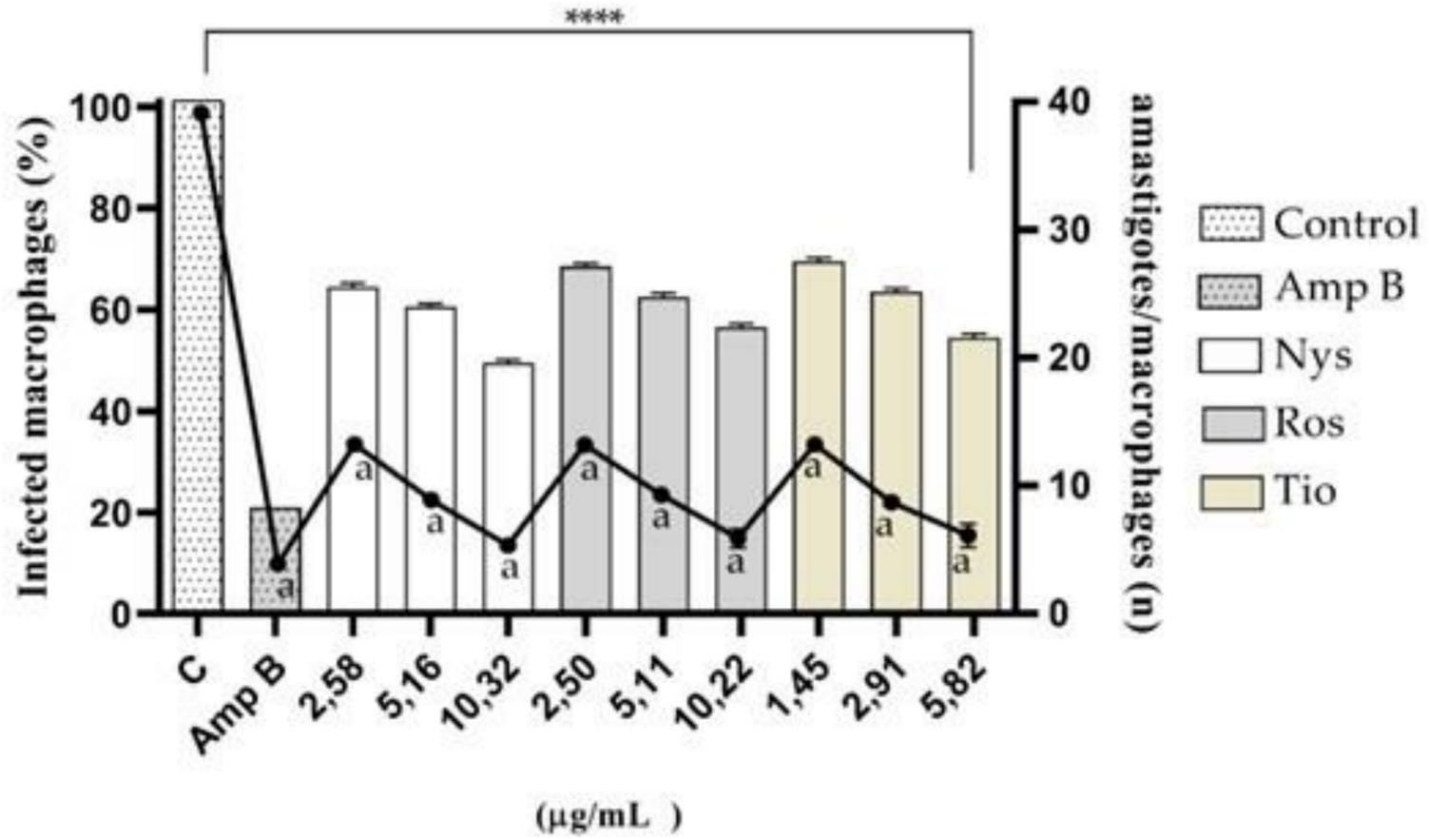
Effects of nystatin (Nys), rosuvastatin (Ros), and tioconazole (Tio) on the infection and infectivity of murine macrophages with *L. major*. The columns correspond to the infection rate and the rows to the number of amastigotes per macrophage. The results represent the mean ± SEM in independent triplicate *p* < 0.05, (a = ****), when compared to control (C), Amp B, and the concentrations tested

**Fig. 2:**
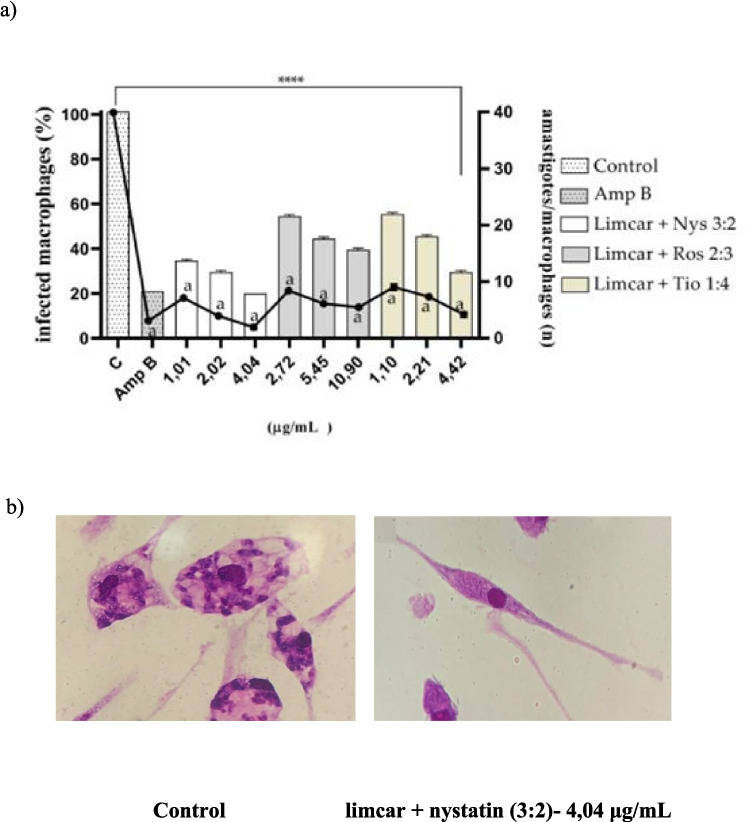
**a** Effects of the associations Limcar + Nys (3:2); Limcar + Ros (2:3) and Limcar + Tio (1:4) in the infection and infectivity of murine macrophages with *L. major*. **b** Macrophages experimentally infected with *L. major* and treated with the combination Limcar + nystatin (3:2). The results represent the mean ± SEM in independent triplicate *p* < 0.05 (a = ****), when compared to control (C), Amp B, and the concentrations tested.

In parallel with these results, the evaluated compounds were able to reduce the number of infected macrophages and the number of amastigotes within them. Groups treated with the drugs Nys, Ros, and Tio at their respective IC_50_ concentrations exhibited similar infection rates of approximately 60% (Fig. 1). Among the combinations, the best results were observed for the following ratios: [Lim-Car (4:1) + Nys] (3:2) with an infection rate of 35%; [Lim-Car (4:1) + Ros] (2:3) with a rate of 50%; and [Lim-Car (4:1) + Tio] (1:4) with a reduction to 55% (Fig. 2a). The untreated control group showed 100% parasitized cells (Fig. 2b).

**Fig. 3:**
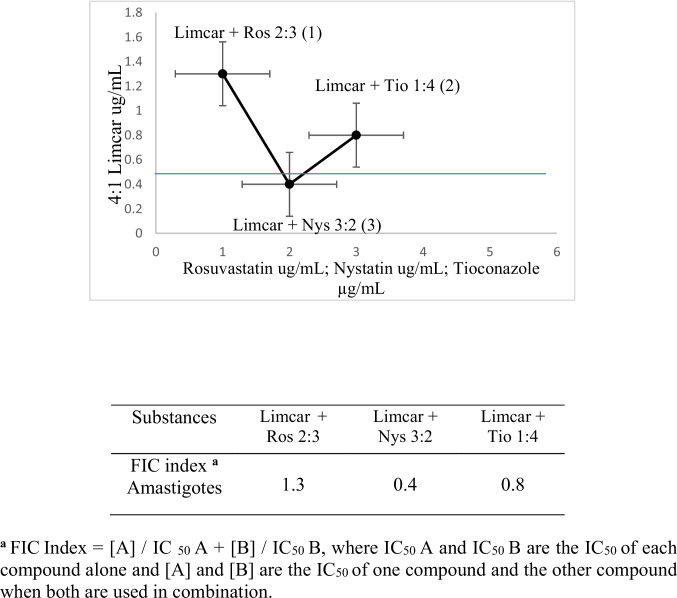
Evaluation of synergism obtained by the fixed proportion method based on IC_50_ values by combining Lim-car monoterpenes with rosuvastatin, nystatin, and tioconazole in *L. major* amastigotes and isobologram obtained

When analyzing the infectivity index, represented by the number of intracellular amastigotes per cell, the untreated control group presented an average of 38 amastigotes/macrophage, considered the survival index. In the group treated with Amp B (0.62 µg/mL), a reduction in the number of parasites to approximately 1.67 amastigotes/macrophage was observed. For groups treated with the drugs, as well as those treated with the combinations, a concentration-dependent reduction in the number of amastigotes was observed (Figs. 1 and 2). Infected cells treated with the combination [Lim-Car (4:1) + Nys] (3:2), at concentrations corresponding to ½ × IC_50_ (1.01 µg/mL), 1 × IC_50_ (2.02 µg/mL), and 2 × IC_50_ (4.04 µg/mL), showed the best results compared to other groups (3.5, 2.0, and 1.5 amastigotes per macrophage), including the outcome observed in the Amp B-treated control group (Fig. 3).

The selectivity index (SI) reflects the cytotoxicity and biological activity of compounds, adapting to the highest active concentration without cellular toxicity due to antileishmanial activity against intracellular amastigote forms in macrophages. The combination [Lim-Car (4:1) + Nys] (3:2) stood out with a selectivity index of 33.60, demonstrating higher selectivity for the *L. major* parasite than for murine macrophages (Table 2).

Given the limitations of current medications, the need for combination therapy involving drugs or bioactive compounds for leishmaniasis treatment has emerged as a more rational approach. This strategy increases drug efficacy, reduces treatment duration and dosage, and minimizes toxicity and drug resistance (Abou-El-Naga et al. 2020).

To assess whether the combination of monoterpenes with drugs inhibiting ergosterol synthesis acted synergistically, the results were subjected to isobologram analysis using the fixed-ratio method to evaluate potential synergy between compounds. The most conservative and widely recommended interpretation for isobolograms in the literature is based on the fractional inhibitory concentration (FIC) index: synergy (FIC ≤ 0.5), antagonism (FIC > 4.0), and additive or indifferent effects (FIC > 0.5–4.0) (Mesquita et al. 2014).

Carvalho et al. (2021) combined limonene and carvacrol in a 4:1 ratio and observed an additive effect with ΣFIC = 0.7, showing antiparasitic activity against promastigote and amastigote forms of *L. major* and lower cytotoxicity for murine macrophages. In our tests, in addition to combining monoterpenes at the same 4:1 ratio, we also combined the drugs Nys, Ros, and Tio and obtained an overall ΣFIC ranging from 1.3 to 0.4 (Table 2). Based on these results, the combination [Lim-Car (4:1) + Nys] (3:2) presented an FIC value < 0.5, demonstrating a synergistic effect against intracellular amastigotes of *L. major*. The other compounds, according to the adopted classification, were not considered synergistic but rather cases of additivity (Fig. 3).

In the evaluation of phagocytic capacity and lysosomal activity, our results showed that in groups treated with the isolated substances and the combinations, there was no increase in lysosomal volume or phagocytic capacity (data not shown). The evaluated substances responded similarly in the nitric oxide (NO) synthesis induction assay, an indicator of macrophage activation through nitrite quantification via macrophage incubation with the compounds. As with the initial macrophage activation results, none of the compounds showed the ability to induce this metabolite. These experimental findings corroborate the mechanism of action of the evaluated drugs, which act by damaging the parasite membrane through their effect on ergosterol, thereby inhibiting parasite growth without inducing cellular activation (Yamamoto et al. 2018).

**Fig. 4:**
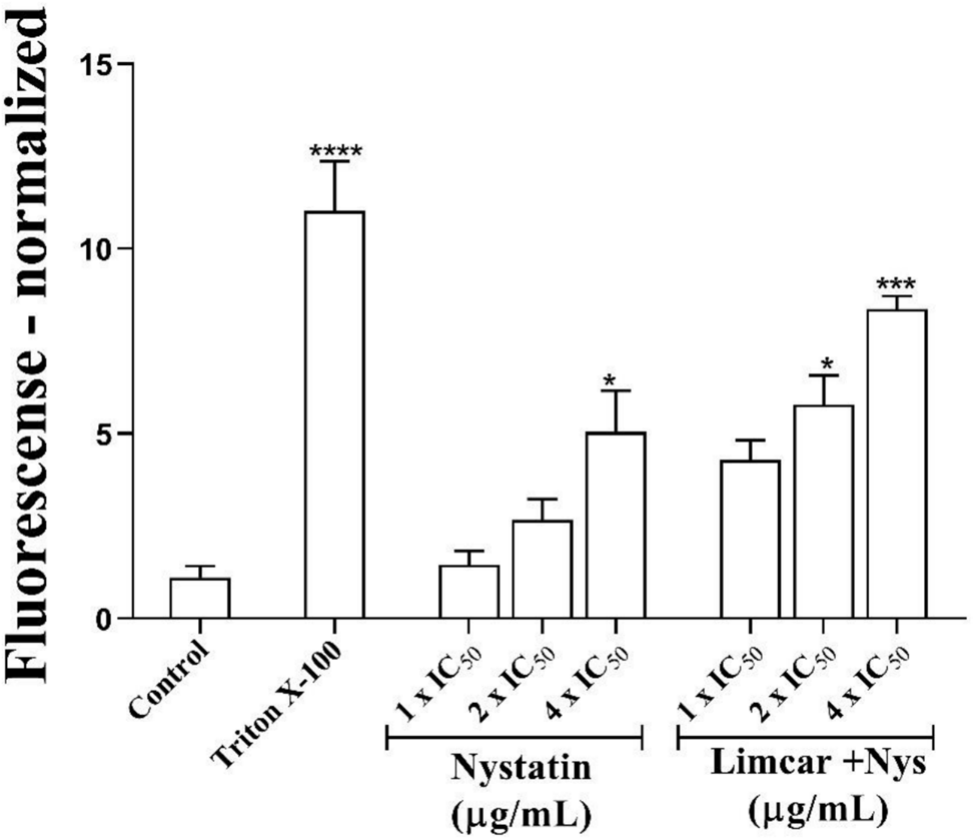
Effects of nystatin and limcar + nystatin combination on the plasma membrane permeability of *Leishmania major* promastigotes. A SYTOX green probe was used. Data represent the normalized fluorescent mean standard error for three independent experiments performed in triplicate. *p < 0.05 vs. control; ***p < 0.001 vs. control

To determine potential damage to the parasite membrane, membrane permeability assays were conducted. A significant increase in permeability (via fluorescence signal) was observed in promastigotes treated with nystatin at 4 × IC_50_ (*p* < 0.001). The combination [Lim-Car (4:1) + Nys] (3:2) showed superior results at 2 × IC_50_ and 4 × IC_50_ (2.02 ± 0.8 µg/mL) concentrations (*p* < 0.001), closely resembling the positive control—promastigotes treated with 0.1% Triton X-100 (Fig. 4).

It has been demonstrated that nystatin and the combination [Lim-Car (4:1) + Nys] (3:2) act directly on the parasite membrane, with the combination intensifying plasma membrane permeability changes compared to the isolated drug. These findings are consistent with experimental evidence showing that monoterpenes such as limonene and carvacrol disrupt the integrity and biophysical properties of Leishmania membranes, leading to increased permeability and structural destabilization. Recent studies on the limonene–carvacrol combination demonstrate marked membrane damage, mitochondrial dysfunction, and modulation of redox metabolism in *Leishmania major*, reinforcing the capacity of monoterpenes to act simultaneously on membrane components and intracellular targets (Carvalho et al. 2021). Additionally, β-ocimene, another monoterpene with structural similarity, has been shown to induce oxidative stress, compromise membrane integrity, and reduce mitochondrial potential in *Leishmania amazonensis* (Sousa et al. 2023). Together, these findings support that monoterpenes can affect multiple biochemical pathways, including redox homeostasis and mitochondrial function, contributing to parasite death through mechanisms that extend beyond simple membrane disruption. This evidence strengthens the rationale for exploring combination therapies that leverage the multitarget action of monoterpenes to enhance leishmanicidal efficacy.

**Fig. 5:**
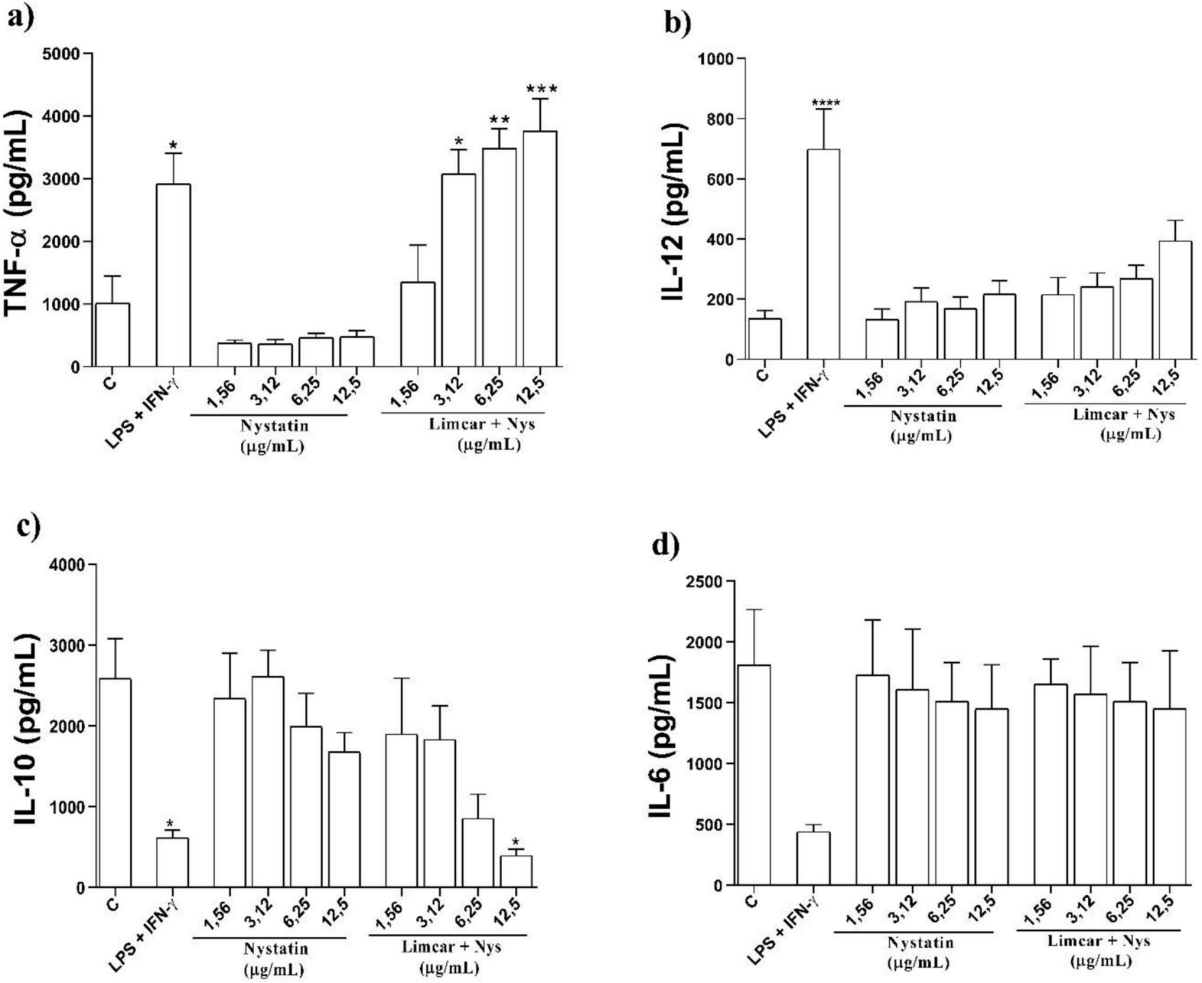
Levels of cytokines TNF-α (**a**), IL-12 (**b**), IL-10 (**c**), and IL-6 (**d**) produced by murine macrophages infected with *Leishmania major* and treated with nystatin and [limcar (4:1) + Nys] (3:2) for 72 h at 37 °C and 5% CO_2_. Results represent means ± standard error for three independent experiments performed in triplicate. *p < 0.5 vs. infected control; **p < 0.01 vs. infected control; ***p < 0.001 vs. infected control; ****p < 0.0001 vs. infected control. C, control; LPS, lipopolysaccharide

Based on the observed results, combination studies have demonstrated potential compared to the compounds evaluated individually, seeking to identify possible targets for the combined compounds. Although macrophage activation was not demonstrated, antileishmanial activity could also be correlated with immunomodulatory effects on cytokine production. As shown in Fig. 5, the combination [Lim-Car (4:1) + Nys] (3:2) induced a host-protective cytokine response, with an increase in TNF-α production (Fig. 5a) and a decrease in IL-10 (Fig. 5c) levels in murine macrophages infected with *L. major*. Regarding IL-6 (Fig. 5d), although a concentration-dependent reduction was observed, this difference was not statistically significant under the evaluated conditions. Similarly, the compounds did not significantly induce IL-12 levels (Fig. 5b).

Our results demonstrated antileishmanial activity targeting the parasite membrane, likely due to the action of monoterpenes and the drugs used on ergosterol. Additionally, superior leishmanicidal effects were observed with the combined substances. It is important to highlight that the evaluated compounds already showed significant advancements, as the assessment of antileishmanial activity using terpenes (including monoterpenes, diterpenes, sesquiterpenes, and triterpenes) revealed numerous mechanisms of action. These include antiproliferative activities, parasite elimination through increased reactive oxygen species and NO production, phosphatidylserine exposure, mitochondrial and DNA damage, iron uptake, autophagy, and the production of pro-inflammatory cytokines such as TNF-α, with this significant increase observed in our study (Rodrigues et al. 2021). Interestingly, nystatin also has the capacity to increase TNF-α levels in mice (Semis et al. 2012). This finding may explain the superiority of the [Lim-Car (4:1) + Nys] (3:2) combination, as it can exert additive effects across multiple biochemical pathways, affecting both the parasite membrane and increasing the expression of Th1-profile cytokines while reducing Th2-profile cytokine levels. This leads to parasite elimination and, consequently, a reduced parasitic load.

The additive effects observed are biologically relevant, as the combination simultaneously disrupts parasite membrane integrity and promotes a Th1-oriented immune response, which is essential for effective parasite control. The increase in TNF-α accompanied by reduced IL-10 supports enhanced macrophage activation and improved intracellular parasite killing. Experimental evidence shows that carvacrol can modulate pro-inflammatory signaling pathways, including NF-κB and ERK1/2, leading to increased production of Th1-associated cytokines and contributing to parasite control mechanisms (Somensi et al. 2019). In addition, antifungal agents targeting sterol pathways, such as nystatin, have been shown to exert immunomodulatory effects that favor a Th1 profile and reduce disease burden in vivo (Semis et al. 2012). Together, these findings reinforce that the combination evaluated in this study acts through complementary biochemical and immunological mechanisms, enhancing its therapeutic potential.

**Fig. 6:**
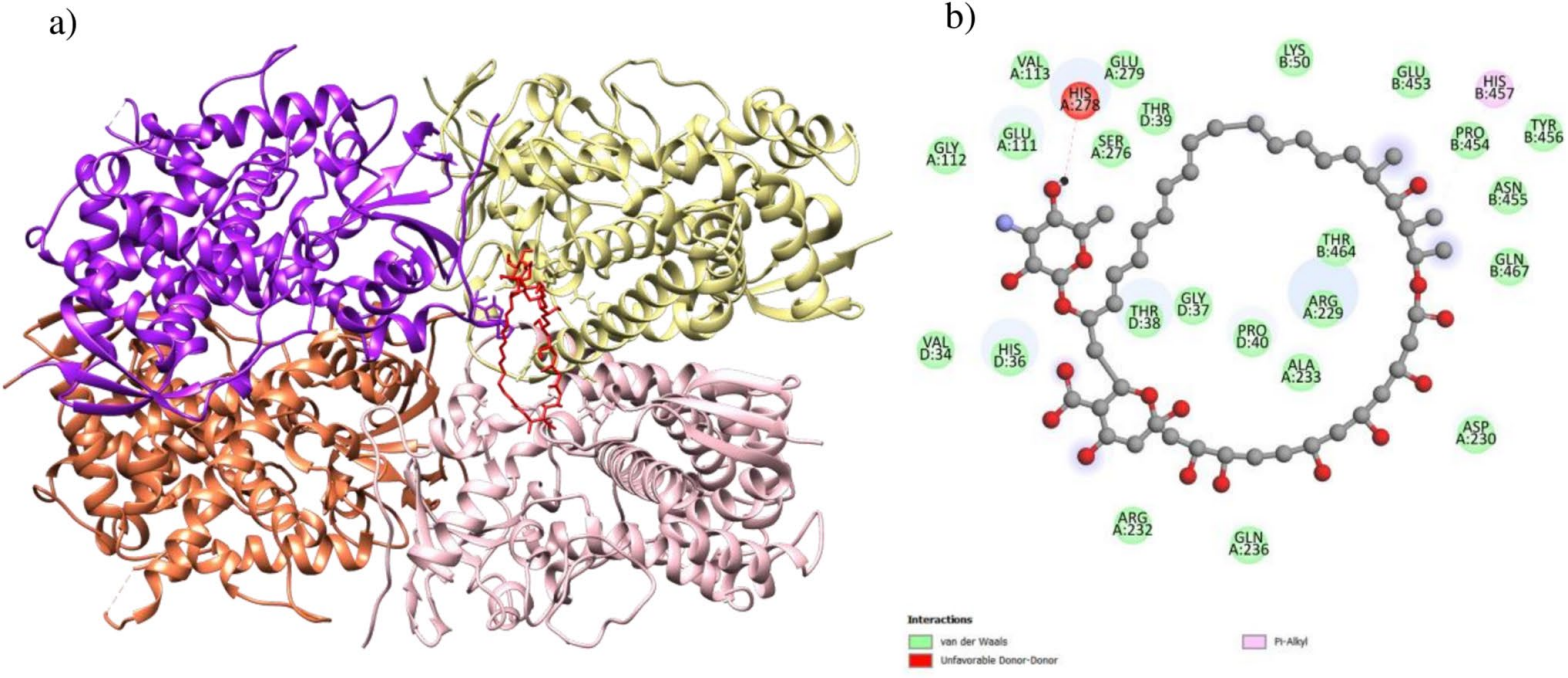
3D molecular docking of the ligand–protein complex with 3L4D (chain A color: khaki; chain B color: pink; chain C color: coral; chain D color: purple) and nystatin (color: red) illustrating the active binding site (**a**) with the respective interactions (**b**)

There are still crucial proteins for parasitic survival that have been highlighted as interesting targets in the development of new therapies. Among these, the enzymes trypanothione reductase (TryR), essential in the detoxification system against oxidative damage induced by the host’s immune system, and sterol 14-alpha demethylase (CYP51), involved in ergosterol biosynthesis and cellular membrane stability of the parasite, were chosen as targets for molecular docking studies, a parameter used to evaluate the energy required to stabilize a molecule in an enzymatic catalytic site (Mukherjee et al. 2020). The results of the interaction between nystatin and the enzymes TryR and CYP51 are presented in Table 3. Optimal binding energy parameters were obtained from the interaction between nystatin and CYP51. Binding affinity was observed with a binding energy of − 8.9 kcal·mol^−1^, as lower binding energy corresponds to better binding (Table 3; Fig. 6).

**Table 3:** Molecular affinity parameters of Nystatin with 2JK6 and 3L4D proteins

Complex (ligand–protein)	ΔG_bind_^a^ (kcal.mol^−1^)	Ligands interactions with residues of proteins^b^
Nistatina-3L4D	− 8.9	Gly A: 112, Glu A: 111, Val A: 113, His A: 278, Ser A: 276, Glu A: 279, Thr D: 39, Lys B: 50, Glu B: 453, Pro B: 454, His B: 457, Tyr B: 456, Asn B: 455, Gln B: 467, Asp A: 230, Gln A: 236, Arg A: 232, His D: 36, Val D: 34, Thr D: 38, Gly D: 37, Pro D: 40, Ala A: 233, Arg A: 229, Thr B: 464
Nistatina-2JK6	− 8.5	Glu B: 18, Trp B: 21, Asn B: 22, Arg A: 472, Thr B: 26, Glu B: 347, Ala B: 343, Arg B: 355, Asn B: 340, Ser A: 470, Ile B: 339, Cys B: 52, Val B: 53, Thr B: 335, Tyr B: 110, Leu B: 17, His A: 461, Gly B: 49, Ser B: 14, Leu A: 399, Phe A: 396, Thr A: 397, Glu A: 466

^a^Power bond in best conformation

^b^Obtained with BIOVIA Discovery Studio Visualizer

In the formation of this complex, interactions were observed with the following amino acids: Gly A:112, Glu A:111, Val A:113, His A:278, Ser A:276, Glu A:279, Thr D:39, Lys B:50, Glu B:453, Pro B:454, His B:457, Tyr B:456, Asn B:455, Gln B:467, Asp A:230, Gln A:236, Arg A:232, His D:36, Val D:34, Thr D:38, Gly D:37, Pro D:40, Ala A:233, Arg A:229, and Thr B:464 within the protein’s active site bound to nystatin.

**Fig. 7:**
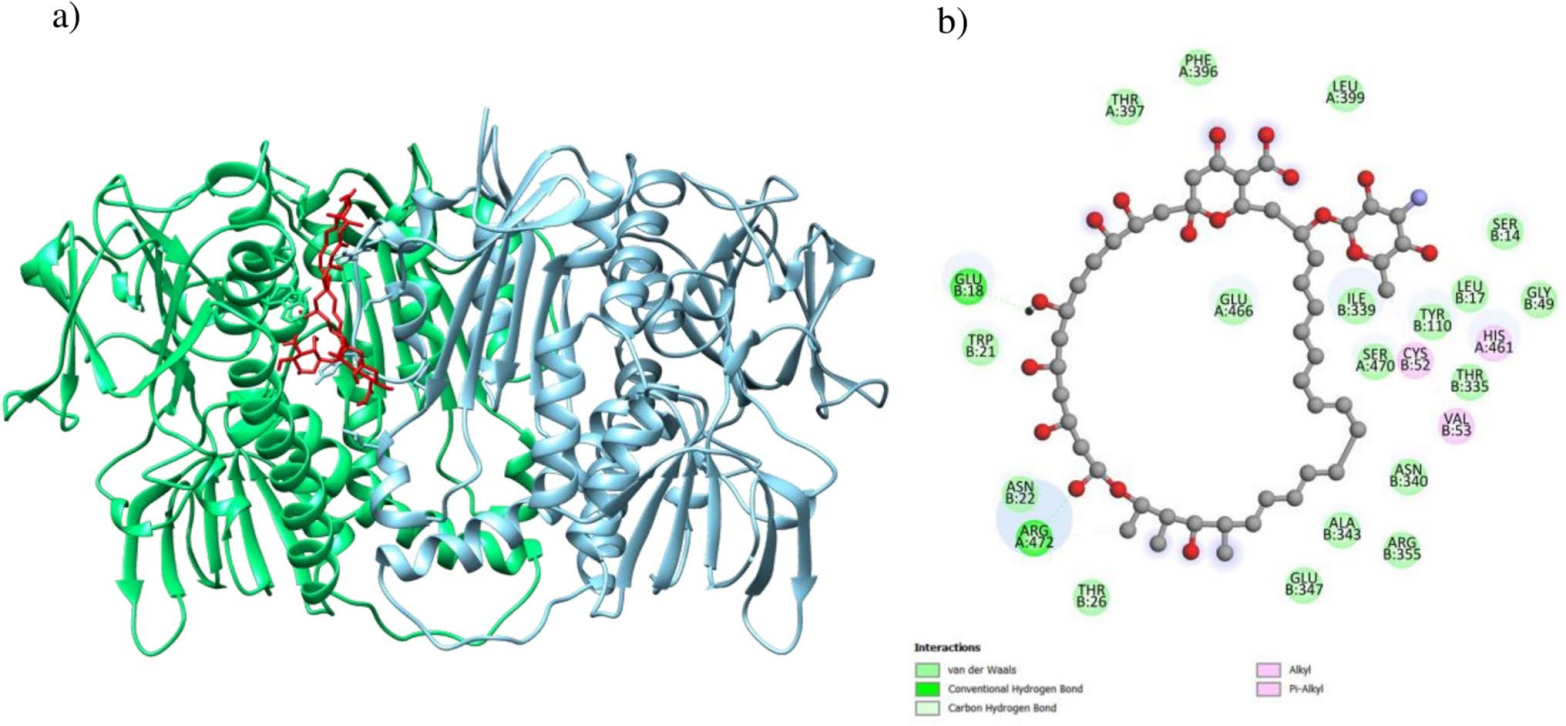
3D molecular docking of the ligand–protein complex with 2JK6 (chain A color: blue; chain B color: green) and nystatin (color: red) illustrating the active binding site (**a**) with the respective interactions (**b**)

Nystatin is a polyene macrolide that acts, in antifungal therapy, as an ergosterol inhibitor, causing various biological alterations. Given that the leishmania parasite also uses this sterol as a membrane component, combined with the molecular docking results showing that the drug demonstrated a higher binding affinity for the CYP51 target (− 8.9 kcal·mol^−1^) and a significant binding value of − 8.5 kcal·mol^−1^ (Table 3; Fig. 7) for TryR, this expands our understanding of the mechanism of action of the combination [Lim-car (4:1) + Nys] (3:2). Carvalho et al. (2021) reported that the best ligands for carvacrol and limonene (− 6.44 and − 6.26 kcal·mol^−1^, respectively) were the TryR target, suggesting that they may be potent inhibitors of TryR and CYP51, crucial enzymes for parasite survival but absent in the human host.

The analysis of the interactions between nystatin and the enzymes CYP51 and TryR revealed the presence of multiple hydrogen bonds, hydrophobic interactions, and polar contacts with critical active-site residues such as Glu, His, Tyr, and Arg (Figs. 6 and 7). This interaction pattern is consistent with experimental studies demonstrating that ligand stability within the CYP51 active site strongly depends on a combination of deep hydrogen-bonding networks and hydrophobic interactions inside the catalytic cavity, which contribute to a more stable and specific binding mode (Mukherjee et al. 2020). Similarly, in silico analyses integrated with biochemical data show that compounds acting on redox-related enzymes in *Leishmania*, such as TryR, also rely on polar and hydrophobic contacts to maintain complex stability and block access of the natural substrate, reinforcing the competitive inhibition potential observed for nystatin (Amlabu et al. 2021). These findings strengthen the interpretation that the interactions detected in the present study are chemically consistent and compatible with mechanisms previously described for ligands exhibiting high affinity toward CYP51 and TryR.

Based on the findings of this study, it is possible to suggest that the combination of monoterpenes (limonene and carvacrol) with nystatin could be proposed for future trials in the treatment of cutaneous leishmaniasis. Investigating synergism through drug combinations can accelerate preclinical and clinical trial processes, with combination therapy representing a significant advance for leishmaniasis treatment. However, further exploration of vital parasite pathways is necessary to understand the potential of combination therapy fully.

## Conclusion

In our study, the combination [Lim-Car (4:1) + Nys] (3:2) was the most effective against *L. major*, with low cytotoxicity and the one that showed the greatest selectivity. Furthermore, it was the only one to show synergism, which potentiated its action. Although the combination was not able to activate macrophages, indirect immunomodulation was evidenced with an increase in TNF-α and a decrease in IL-10 and IL-6, as well as changes in the integrity of the plasma membrane and a greater susceptibility to the enzyme 14-alpha demethylase in a molecular docking study. Therefore, we highlight the importance of the combination of drugs and natural products as a therapeutic potential for in vivo trials and the perspective of repositioning nystatin as an alternative for leishmaniasis therapy.

## Data Availability

Data can be available under request to the authors.

## Abbreviations

Amph B: Amphotericin B
DMSO: Dimethyl sulfoxide
FBS: Fetal bovine serum
Lim-Car: Limonene and carvacrol combination
MTT: 3-(4.5-Dimethylthiazol-2-yl)-2.5-diphenyltetrazolium bromine
NYS: Nystatin
NO: Nitric oxide
ROS: Rosuvastatin
PBS: Phosphate buffered saline
TIO: Tioconazole
TYR: Trypanothione reductase
CYP51: 14-Alpha demethylase
